# Gliotoxin potentiates osteoblast differentiation by inhibiting nuclear factor-κB signaling

**DOI:** 10.3892/mmr.2015.3524

**Published:** 2015-03-20

**Authors:** GUANGYE WANG, XIAOHAI ZHANG, BAOQING YU, KE REN

**Affiliations:** 1 The Third Department of Orthopaedics, Wuhu Second People’s Hospital, Wuhu, Anhui 241000, P.R. China; 2 Department of Orthopedics, Shanghai Pudong Hospital, Fudan University Pudong Medical Centre, Shanghai 201399, P.R. China; 3 Department of Orthopedics, Nanjing General Hospital of Nanjing Military Command Region, Nanjing, Jiangsu 210002, P.R. China

**Keywords:** gliotoxin, osteoblast differentiation, tumor necrosis factor-α, nuclear factor-κB

## Abstract

The differentiation of pluripotent mesenchymal stem cells to mature osteoblasts is crucial for the maintenance of the adult skeleton. In rheumatic arthritis, osteoblast differentiation is impaired by the overproduction of cytokine tumor necrosis factor (TNF)-α. It has been demonstrated that TNF-α is able to inhibit osteoblast differentiation through the activation of nuclear factor (NF)-κB signaling. As a result of the critical role of TNF-α and NF-κB in the pathogenesis of bone-loss associated diseases, these factors are regarded as key targets for the development of therapeutic agents. In the current study, the role of the NF-κB inhibitor gliotoxin (GTX) in the regulation of osteoblast differentiation was evaluated. The non-toxic GTX doses were determined to be ≤3 *μ*g/ml. It was revealed that GTX was able to block TNF-α-induced inhibition of osteoblast differentiation, as indicated by alkaline phosphatase (ALP) activity and ALP staining assays, as well as the expression levels of osteoblast-associated genes *Col I, Ocn, Bsp, Runx2, Osx* and *ATF4*. Additionally, it was identified that gliotoxin directly promoted bone morphoge-netic protein-2-induced osteoblast differentiation. GTX was found to inhibit the accumulation of NF-κB protein p65 in the nucleus and reduce NF-κB transcriptional activity, suggesting that GTX potentiated osteoblast differentiation via the suppression of NF-κB signaling.

## Introduction

Osteoblasts are derived from pluripotent mesenchymal stem cells (MSCs), which are capable of differentiation into multiple cell types, including chondrocytes, osteoblasts and adipocytes (1). Embryonic skeletal development and adult bone maintenance require efficient osteoblast differentiation from MSCs, and mature osteoblasts are able to continuously synthe-size bone matrix to build bone as required (2). Appropriate osteoblast differentiation requires stimulation from extracellular signaling and the expression of osteoblast transcription factors (3). Stimulation from the cell signals, including bone morphogenetic proteins (BMPs), Wingless-ints (Wnts) and Notch, are considered to be critical for osteoblast differentiation from MSCs (3). It has been suggested that several transcription factors, including Runt-related transcription factor 2 (*Runx2*), Osterix (*Osx*) and activating transcription factor 4 (*ATF4*), are major regulators of osteoblast differentiation (4).

Inflammatory factors may impair the differentiation of mesenchymal precursors into mature osteoblasts (5). In rheumatoid arthritis (RA), the overproduction of tumor necrosis factor-α (TNF-α) in the inflammatory joints results in local bone loss, which is due to the reduction in osteoblast differentiation (6,7). In the case of estrogen deficiency, TNF-α is overproduced by activated T-cells (8,9), resulting in the inhibition of osteoblast differentiation and inducing the development of osteoporotic diseases.

To the best of our knowledge, the first *in vitro* inhibitory effect of TNF-α on osteoblast differentiation was identified by Canalis (10) in 1987. Further investigations revealed that TNF-α inhibited the differentiation of fetal calvarial precursor cells to mature osteoblasts *in vitro* (11). *In vivo* studies of TNF-α or p55 receptor gene knockout mice indicated that TNF-α reduced mouse maximum peak bone mass and inhibited osteoblastic bone formation through the downstream nuclear factor-κB (NF-κB) signaling pathway (12). NF-κB signaling was demonstrated to have an endogenous inhibitory effect on osteoblastic bone formation, and osteoblast-specific inactivation of NF-κB signaling rescued the bone mass in an overiectomized mouse model (13). In conclusion, TNF-α and its downstream NF-κB signaling have a critical role in the suppression of osteoblast differentiation and may contribute to adult bone loss.

TNF-α has a complex cell signaling process and influences multiple cellular activities. TNF-α binds to two receptors, TNF receptor type I (p55/60) and TNF receptor type II (p75/80) (14). TNF-α activates various downstream signals, including mitogen activated protein kinase, cell death signals and NF-κB signaling (15). NF-κB signaling was found to exert multiple effects on bone tissue maintenance (16). NF-κB is essential for the differentiation and maturation of osteoclasts, which are required in bone resorption (17). In addition, NF-κB signaling suppresses osteoblast differentiation and bone formation (13). The appropriate balance between bone resorption and bone formation determines the precise levels of bone maintenance in the adult skeleton (18). The activation of NF-κB signaling requires the degradation of the inhibitory protein IκBα, which binds the NF-κB complex and prevents its translocation to the nucleus. The degradation of IκBα facilitates the entrance of the NF-κB complex to the nucleus and induces the subsequent transcriptional activity (19).

Gliotoxin (GTX) is a secondary metabolite, derived from numerous fungi (20–22). GTX has been demonstrated to have antibacterial, antiviral and immunosuppressant activities (23). GTX is considered an NF-κB signal inhibitor, and functions by blocking IκBα degradation, thereby preventing the NF-κB complex from entering the nucleus, which subsequently inhibits NF-κB complex-induced transcriptional activity (24,25). Due to its inhibition of NF-κB signaling, GTX was later found to be a potential anti-inflammatory agent for the treatment of immune glomerulonephritis (26). The activation of NF-κB signaling may prevent cell apoptosis in certain types of cell; therefore, it is considered that NF-κB inhibitor GTX is able to facilitate cell apoptosis (27). For example, GTX was found to enhance radiation-induced apoptosis through NF-κB signaling inhibition (28). The present study aimed to explore the potential role of GTX in the inhibition of NF-κB signaling in C2C12 mesenchymal cells, and its potential function in the regulation of osteoblast differentiation.

## Materials and methods

### Cell cultures and the induction of osteoblast differentiation

The C2C12 mesenchymal cell line was obtained from American Type Culture Collection (Manassas, VA, USA). The monolayer culture was maintained in growth medium containing Dulbecco’s modified Eagle’s medium (Invitrogen Life Technologies, Carlsbad, CA, USA), supplemented with 10% fetal bovine serum (FBS), 50 U/ml penicillin and 50 mg/ml streptomycin (all obtained from Hyclone, Thermo Fisher Scientific, Logan, UT, USA). The cultures were incubated in a humidified atmosphere at 37°C with 5% CO_2_. To determine the function of GTX for protecting osteoblast differentiation from inhibition by TNF-α, C2C12 cells were divided into various groups. The BMP-2 group was treated with 200 ng/ml recombinant human BMP-2 (R&D Systems, Rockville, MD, USA); the TNF-α alone group was treated with 10 ng/ml TNF-α (Peprotech, Inc., Rocky Hill, NJ, USA); the BMP-2 + TNF-α group was treated with a combination of 200 ng/ml recombinant human BMP-2 and 10 ng/ml TNF-α; the GTX group was treated with a combination of 200 ng/ml recombinant human BMP-2 and 10 ng/ml TNF-α, as well as the indicated quantity of GTX simultaneously. Cells were incubated in a humidified atmosphere at 37°C and 5% O_2_ for 72 h. To examine the effect of GTX in modulating BMP-2-induced osteoblast differentiation, C2C12 cells were divided into different groups. The BMP-2 group was treated with 200 ng/ml recombinant human BMP-2; the GTX alone group was treated with 1 *μ*g/ml GTX; the BMP-2 + GTX group was treated with a combination of 200 ng/ml recombinant human BMP-2 and 1 *μ*g/ml GTX. Cells were incubated in a humidified atmosphere at 37°C and 5% CO_2_. For the ALP activity assay, cells were incubated for 2 days. For the polymerase chain reaction (PCR) assay, cells were incubated for 3 days.

### RNA isolation and PCR

The total RNA of C2C12 cells was isolated using TRIzol reagent (Invitrogen Life Technologies) according to the manufacturer’s instructions. For reverse transcription, 2 *μ*g total RNA was used and mixed with 5 *μ*M OligodT, 2 *μ*l 10X RT buffer, 0.5 mM dNTP mix, 10 units of RNase inhibitor, 100 units of reverse transcriptase and nuclease-free water was added to a final 20 *μ*l volume. The reaction solution was incubated in an Eppendorf thermocycler (Mastercycler nexus X2; Eppendorf GmbH, Hamburg, Germany) at 42°C for 1 h and 72°C for 10 min. All the reagents were purchased from Promega Corp. (Madison, WI, USA). PCR was performed using an ABI 7900HT system (Applied Biosystems Life Technologies, Foster City, CA, USA) with SYBR1 Premix^Ex^ Taq™ (Takara, Dalian, China), according to the manufacturer’s instructions. *GAPDH* was used as the internal control. Each sample was analyzed in triplicate. The primer sequences for C2C12 cells used in the present study were as follows: *GAPDH* forward, 5′-GACTTCAACAGCAACTCCCAC-3′ and reverse, 5′-TCCACCACCCTGTTGCTGTA-3′; type I collagen (*Col I*) forward, 5′-GAGCTGGTGTAATGGGTCCT-3′, and reverse, 5′-GAGACCCAGGAAGACCTCTG-3′; bone sialoprotein (*Bsp*) forward, 5′-CAGGGAGGCAGTGACTCTTC-3′ and reverse, 5′-AGTGTGGAAAGTGTGGCGTT-3′; Osteocalcin (*Ocn*) forward, 5′-AAGCAGGAGGGCAATAAGGT-3′ and reverse, 5′-TTTGTAGGCGGTCTTCAAGC-3′; *Runx2* forward, 5′-GACTGTGGTTACCGTCATGGC-3′ and reverse, 5′-ACTTGGTTTTTCATAACAGCGGA-3′; *ATF4* forward, 5′-CCTGAACAGCGAAGTGTTGG-3′ and reverse, 5′-TGGAGAACCCATGAGGTTTCAA-3′; *Osx* forward, 5′-GGAAAGGAGGCACAAAGAAGC-3′ and reverse 5′-CCCCTTAGGCACTAGGAGC-3′.

### Western blotting

Cells were lysed on ice for 30 min in lysis buffer (Thermo Fisher Scientific), which was comprised of 50 mM Tris-HCl (pH 7.4), 150 mM NaCl, 1% Nonidet P-40 and 0.1% SDS supplemented with protease inhibitors (10 mg/ml leupeptin, 10 mg/ml pepstatin A and 10 mg/ml aprotinin). Protein content was measured with Pierce bicinchoninic acid (BCA) reagent (Pierce Biotechnology, Inc., Rockford, IL, USA) according to the manufacturer’s instructions. Cytosolic and nuclear fractions were prepared with a Nuclear and Cytoplasmic Protein Extraction kit (Beyotime Institute of Biotechnology, Shanghai, China), according to the manufacturer’s instructions. For western blot analysis, 20–40 mg of sample was resolved on 12% SDS-PAGE and electro-transferred onto nitrocellulose membranes (Whatman, Piscataway, NJ, USA). Anti-GAPDH, anti-p65 and anti-Lamin B antibody (all from Santa Cruz Biotechnology, Inc., Dallas, TX, USA) were used at a 1:1,000 dilution and incubated with the protein samples at 4°C overnight. The anti-GAPDH antibody (cat. no. sc-365062) was a mouse monoclonal IgG, raised against amino acids 1-335 representing a full length GAPDH of human origin. The anti-p65 antibody (cat. no. sc-8008) was a mouse monoclonal IgG antibody, raised against amino acids 1-286 mapping at the N-terminus of p65 of human origin. The anti-Lamin B antibody (cat. no. sc-365214) was used to determine the loading of nucleus protein level and was a mouse monoclonal IgG, raised against amino acids 559–584 at the C-terminus of lamin B1 of human origin. The goat anti-mouse IgG horseradish peroxidase secondary antibody (cat. no. sc-2031; Santa Cruz Biotechnology, Inc) was used at a 1:2,000 dilution. The antigen-antibody complexes were visualized using an enhanced chemiluminescence detection system (EMD Millipore, Billerica, MA, USA) according to the manufacturer’s instructions.

### Alkaline phosphatase (ALP) activity and staining

C2C12 cells were treated with 200 ng/ml BMP-2 and/or 10 ng/ml TNF-α for 72 h at 37°C. The cultured C2C12 cells were rinsed three times with ice-cold PBS, scraped from the dishes and suspended in double distilled H_2_O, prior to three cycles of freezing and thawing. ALP activity was determined using an Orion™ AquaMate 7000 Vis spectrophotometer (Thermo Fisher Scientific) at 405 nm using *p*-nitrophenyl phosphate (pNPP; Sigma-Aldrich) as the substrate. A 50 ml cell sample was mixed with 50 ml pNPP (1 mg/ml) in 1 M diethanolamine buffer (Sigma-Aldrich) supplemented with 0.5 mM MgCl_2_ (pH 9.8; Sigma-Aldrich) and incubated at 37°C for 15 min on a bench shaker. The reaction was stopped by the addition of 200 ml 2 M NaOH per 200 ml reaction mixture. Total protein content was determined by the BCA method, using a BCA protein assay kit (Pierce Biotechnology, Inc.). ALP activity was presented as fold-changes in activity over the normal control group at the respective time-points. All experiments were conducted in triplicate. For ALP staining, C2C12 cells were rinsed three times with PBS (Sigma-Aldrich) and fixed with 4% paraformaldehyde (Sigma-Aldrich) for 10 min at 4°C. The fixed cells were subsequently soaked in 0.1% naphthol AS-MX phosphate (Sigma-Aldrich) and 0.1% fast red violet LB salt (Sigma-Aldrich) in 56 mM 2-amino-2-methyl-1,3-propanediol (pH 9.9; Sigma-Aldrich) for 10 min at room temperature, washed with PBS and observed under a digital camera (Sony DSC-HX50; Sony Corp., Tokyo, Japan).

### Plasmid transfection and luciferase activity assays

The pGL4.32[luc2P/NF-κB-RE/Hygro] vector plasmids were transfected into C2C12 cells for the determination of NF-κB activity. pRL *Renilla* luciferase (Rluc) control reporter vector plasmids were co-transfected into C2C12 cells with pGL4.32[luc2P/NF-κB-RE/Hygro] vector plasmids as an inner control. The two plasmids were purchased from Promega Corp. and were transfected into cells using Lipofectamine^®^ 2000 (Invitrogen Life Technologies). C2C12 cells were seeded at 0.5×10^5^ cells/well in a 24-well plate in complete medium and were stimulated with 200 ng/ml BMP-2, 10 ng/ml TNF-α and/or 1 *μ*g/ml GTX, 24 h after plasmid transfection. Cells were incubated at 37°C and harvested 48 h later. Luciferase activities were measured using a dual luciferase system (Promega Corp.).

### Immunofluorescence confocal microscopic assay for p65 localization

C2C12 cells were treated with 200 ng/ml BMP-2, 10 ng/ml TNF-α and/or 0.6 *μ*g/ml GTX simultaneously. Following the incubation at 37°C for 1 h, cells were washed twice with PBS and fixed with 4% paraformaldehyde for 20 min. The fixed cells were incubated with primary anti-p65 antibody (1:100; Santa Cruz Biotechnology, Inc.) overnight at 4°C and subsequently incubated with fluorescent secondary antibody for 1 h at room temperature. The anti-p65 antibody (cat. no. sc-8008) was a mouse monoclonal IgG antibody, raised against amino acids 1-286 mapping at the N-terminus of p65 of human origin. The goat anti-mouse IgG-Atto 488 antibody was used as the secondary antibody (cat. no. 62197; Sigma-Aldrich). DAPI was used to stain the nucleic DNA of the cells. The stained cells were analyzed with a Leica TCS SP5 confocal laser scanning microscope (Leica Microsystems GmbH, Wetzlar, Germany).

### MTT and caspase-3 activity assays

Cell viability was determined via MTT assay. MTT was purchased from Sigma-Aldrich. C2C12 cells were treated with various doses of GTX (0, 0.1, 0.5, 1, 2, 3, 4 or 5 *μ*g/ml) and the normal control group was treated with dimethyl sulfoxide. Cells were incubated for 24 or 72 h at 37°C. Subsequently, the culture medium was removed and the cells were washed three times with PBS. Cells were treated with 5 mg/ml MTT salts and incubated at 37°C for 2 h. Absorbance at 570 nm was measured using the Orion™ AquaMate 7000 Vis spectrophotometer (Thermo Fisher Scientific). Apoptosis was characterized by a caspase-3 activity assay, using a caspase fluorescent assay kit (BD Biosciences, San Jose, CA, USA) according to the manufacturer’s instructions C2C12 cells were treated with various doses of GTX (0, 0.1, 0.5, 1, 2, 3, 4 or 5 *μ*g/ml) and the normal control group was treated with DMSO. Cells were incubated for 24 or 72 h at 37°C. The culture medium was removed and cells were washed three times with PBS. Subsequently, cells were lysed on ice for 30 min in lysis buffer (Thermo Fisher Scientific). Cell lysate (50 *μ*l) was mixed with 0.2 ml 1X HEPES buffer and 5 *μ*l reconstituted Ac-DEVD-AMC, incubated at 37°C for 1 h. The AMC liberated from Ac-DEVD-AMC was measured using the Orion™ AquaMate 7000 Vis spectrophotometer (Thermo Fisher Scientific) at an excitation wavelength of 380 nm and an emission wavelength range of 420–460 nm. Caspase-3 activity was presented as fold changes over the normal control group at the respective time points.

### Statistical analysis

Each assay was performed ≥3 times. Each testing group contained a minimum of three samples (n≥3). Values are expressed as the mean ± standard deviation. SPSS software version 22.0 (IBM, Armonk, NY, USA) was used for statistical analysis. Student’s t-test was used to test statistical significance between two groups. For the comparison of more than two groups, one-way analysis of variance was used to determine which groups were significantly different. P<0.05 was considered to indicate a statistically significant difference.

## Results

### TNF-α inhibits C2C12 cell differentiation into osteoblasts

**Figure 1:**
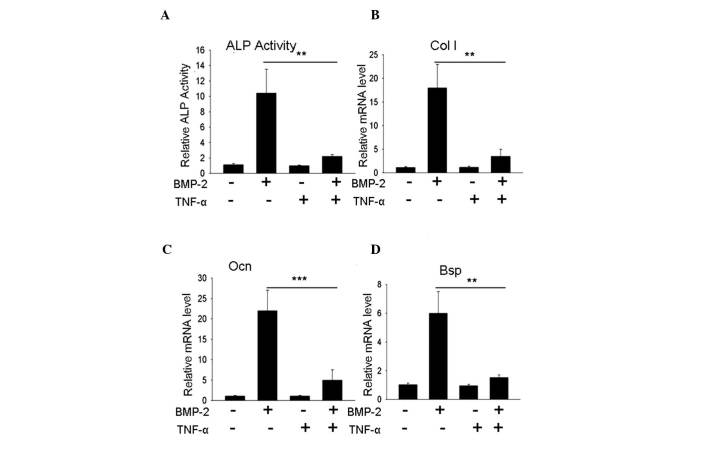
TNF-α inhibits BMP-2-induced mesenchymal C2C12 cell differentiation to osteoblasts. (A) ALP activity assay. C2C12 cells treated with 200 ng/ml BMP-2 revealed an increase in ALP activity; however, in cells treated with a combination of 200 ng/ml BMP-2 and 10 ng/ml TNF-α, the elevation of ALP activity was inhibited. (B–D) Gene expression assays for *Col I*, *Ocn* and *Bsp* indicated that the BMP-2-induced increase in gene expression was inhibited by 10 ng/ml TNF-α. ^**^P<0.01, ^***^P<0.001, n>3. TNF-α, tumor necrosis factor-α; BMP-2, bone morphogenetic protein-2; ALP, alkaline phosphatase; NC, normal control; mRNA, messenger RNA.

As previously reported, TNF-α inhibited the differentiation of mesenchymal precursor cells into osteoblasts. In the present study, whether TNF-α was able to inhibit osteoblast differentiation in a C2C12 cell system was examined. Following three days of treatment, the alkaline phosphatase (ALP) activity of the C2C12 cells was evaluated: The group treated with 200 ng/ml BMP-2 exhibited significantly elevated ALP activity levels compared with the normal control group; whereas, the group treated with a combination of 200 ng/ml BMP-2 and 10 ng/ml TNF-α exhibited a significantly lower level of ALP activity compared with the BMP-treated group (P<0.01), with a value almost equal to that of the normal control group (Fig. 1A). The examination of other osteoblast specific marker genes, including *Col I*, *Ocn* and *Bsp*, indicated that the expression levels of these genes were significantly inhibited (P<0.01, P<0.001; Fig. 1B-D), which was consistent with the results of previous studies. TNF-α effectively inhibited BMP-2-induced osteoblast differentiation in the C2C12 cell system.

### GTX blocks the inhibition of osteoblast differentiation by TNF-α, as indicated by ALP activity and extracellular matrix gene expression

**Figure 2:**
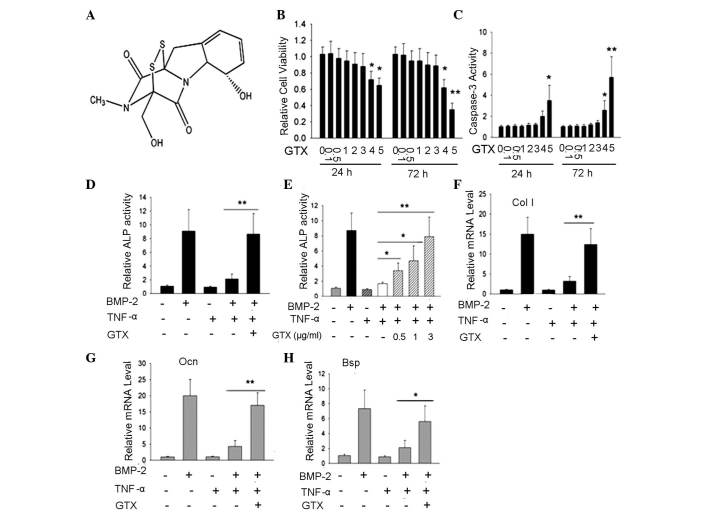
GTX protects osteoblast differentiation against inhibition from TNF-α in C2C12 cells; evidence from ALP activity, as well as *Col I*, *Ocn* and *Bsp* gene expression.(A) Chemical structure of GTX. (B) MTT cell viability assay suggested that, in the groups treated with 4 or 5 *μ*g/ml GTX for 24 or 72 h, cell viability rate was significantly decreased, compared with that of the DMSO-treated control group. (C) Caspase-3 activity assay indicated that following 4 or 5 *μ*g/ml GTX treatment for 24 h or 72 h, caspase-3 activity was markedly enhanced, compared with that of the DMSO control group. (D and E) ALP activity assays performed following 72 h of culture indicated the protective effect of GTX for osteoblast differentiation. (D) TNF-α inhibition of BMP-2-induced ALP activity was blocked by treatment with 1 *μ*g/ml GTX. (E) GTX protected ALP activity in a dose-dependent manner. (F–H) The protective effects of GTX on osteoblast differentiation were determined via analysis of gene expression of *Col I*, *Ocn* and *Bsp*. ^*^P<0.05, ^**^P<0.01, n>3. GTX, gliotoxin; TNF-α, tumor necrosis factor-α; DMSO, dimethyl sulfoxide; ALP, alkaline phosphatase; BMP-2, bone morphogenetic protein-2.

Significant inhibition of NF-κB activity may lead to cell apoptosis; therefore, the appropriate doses for C2C12 cell treatment were determined. C2C12 cells were treated with various doses of GTX (0, 0.1, 0.5, 1, 2, 3, 4 or 5 *μ*g/ml; Fig. 2A) and cell viability was detected following 24 and 72 h of incubation using an MTT assay. Compared with the control group, doses of 0.1–3 *μ*g/ml GTX did not influence cell viability; however, 4 or 5 *μ*g/ml GTX led to a significant reduction in viable cell numbers (P<0.05, P<0.01; Fig. 2B). In addition, the results of a caspase-3 activity assay indicated a significant increase in the levels of apoptosis in the 4 or 5 *μ*g/ml GTX-treated C2C12 cells; however, there were no notable changes in the groups treated with 0.1–3 *μ*g/ml GTX (Fig. 2C). Subsequently, whether GTX influenced the inhibition of osteoblast differentiation by TNF-α was examined. Significant inhibition of ALP activity was observed in the group treated with 200 ng/ml BMP-2 and 10 ng/ml TNF-α compared with BMP-2-treatment alone; however, ALP activity was rescued by the addition of 1 *μ*g/ml GTX (Fig. 2D). Further investigation indicated that the rescued effect was GTX dose-dependent (Fig. 2E). To further confirm that GTX was able to rescue osteoblast differentiation, the expression of three other osteoblast marker genes, *Col I*, *Ocn* and *Bsp*, were evaluated by PCR analysis. The gene expression results demonstrated that when compared with the TNF-α inhibition group, the expression levels of these three genes were rescued by 1 *μ*g/ml GTX (Fig. 2F-H, respectively), (P<0.01, *Col I* and *Ocn*; P<0.05, *Bsp*).

### GTX blocks the inhibition of osteoblast differentiation by TNF-α, as indicated by ALP staining and osteoblast tran- scription factor gene expression

**Figure 3:**
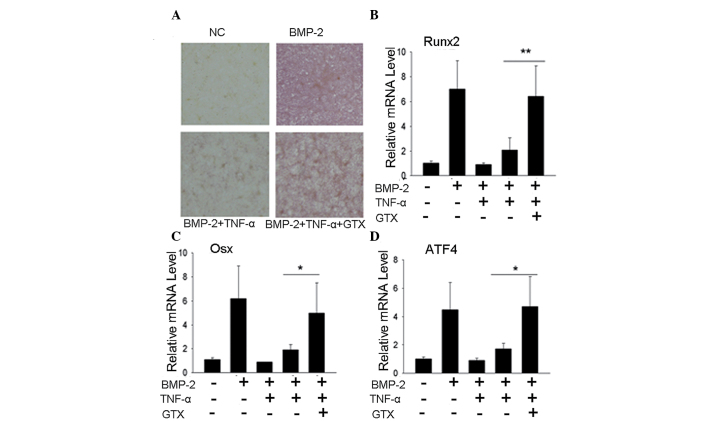
GTX protects BMP-2-induced osteoblast differentiation against inhibition by TNF-α; evidence from ALP staining and gene expression levels of transcription factors. (A) ALP staining assay revealed that 1 *μ*g/ml GTX protected ALP activity against TNF-α-induced inhibition. (B–D) GTX (1 *μ*g/ml) rescued the gene expression of *Runx2*, *Osx* and *ATF4* following TNF-α-induced inhibition. ^*^P<0.05, ^**^P<0.01, n>3. GTX, gliotoxin; TNF-α, tumor necrosis factor-α; ALP, alkaline phosphatase; mRNA, messenger RNA; BMP-2, bone morphogenetic protein-2.

To further confirm that GTX was able to prevent the inhibition of osteoblast differentiation by TNF-α, the expression levels of other genes characteristic of osteoblast differentiation were evaluated. An ALP staining assay revealed that the group treated with 1 *μ*g/ml GTX exhibited a significantly elevated ALP level when compared with that of the TNF-α group (Fig. 3A). PCR results for transcription factor expression indicated a similar effect. The expression levels of *Runx2*, *Osx* and *ATF4* were markedly higher in the 1 *μ*g/ml GTX treatment group compared with those of the TNF-α group (Fig. 3B-D, respectively), (P<0.01, *Runx2*; P<0.05, *Osx* and *ATF4*).

### GTX promotes BMP-2-induced osteoblast differentiation of C2C12 cells

**Figure 4:**
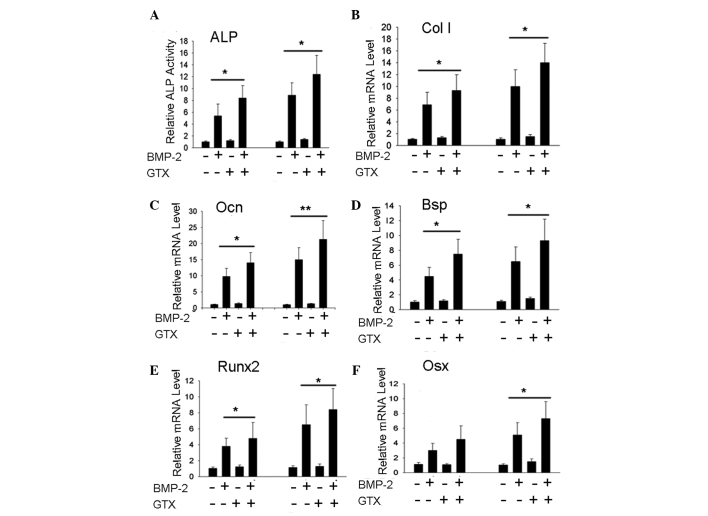
GTX promotes BMP-2-induced osteoblast differentiation in C2C12 cells. (A) BMP-2-induced ALP activity was enhanced by 1 *μ*g/ml GTX treatment. (B–F) Gene expression levels of *Col I*, *Ocn*, *Bsp, Runx2* and *Osx* were found to be enhanced by treatment with 1 *μ*g/ml GTX + BMP-2, compared with those following treatment with BMP-2 alone. For the ALP activity assay, cells were incubated for 2 days and for the polymerase chain reaction assay, cells were incubated for 3 days. ^*^P<0.05, ^**^P<0.01, n>3. GTX, gliotoxin; BMP-2, bone morphogenetic protein-2; ALP, alkaline phosphatase; mRNA, messenger RNA.

Since NF-κB signaling was identified to have endogenous inhibitory effects on osteoblast differentiation, it was hypothesized that blockage of NF-κB signaling may promote osteoblast differentiation and bone formation. Whether GTX had effects on BMP-2-induced C2C12 osteoblast differentiation was therefore evaluated. The results indicated that 1 *μ*g/ml GTX was able to promote ALP activity (Fig. 4A) and expression levels of the extracellular matrix genes *Col I*, *Ocn* and *Bsp* (Fig. 4B–D), as well as the transcription factor genes *Runx2* and *Osx* (Fig. 4E and F; P<0.05, P<0.01).

### GTX modulates C2C12 osteoblast differentiation via NF-κB signal inhibition

**Figure 5:**
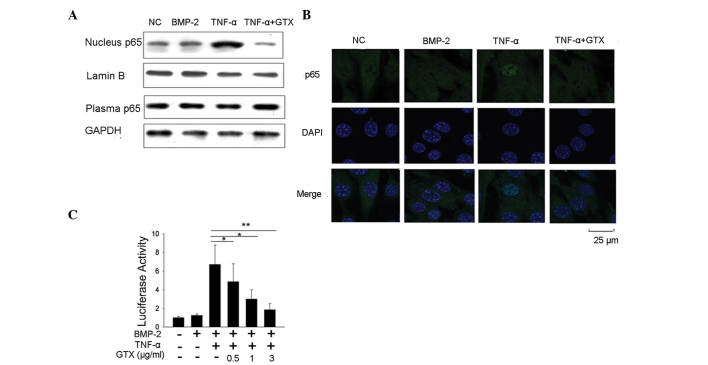
GTX influences osteoblast differentiation via the inhibition of NF-κB signaling in C2C12 cells. (A) Western blot analysis and (B) immunofluorescence confocal microscopic assay revealed that treatment with 10 ng/ml TNF-α induced the aggregation of NF-κB protein p65 in the nucleus; however, this effect was abrogated following 1 *μ*g/ml GTX treatment. (C) Luciferase assay indicated that the activation of NF-κB transcriptional activity induced by BMP-2 + TNF-α treatment was abrogated by GTX treatment in a dose-dependent manner. ^*^P<0.05, ^**^P<0.01, n>3. GTX, gliotoxin; NF-κB, nuclear factor-κB; TNF-α, tumor necrosis factor-α; BMP-2, bone morphogenetic factor-2; NC, normal control.

As indicated by our above results, GTX was able to block TNF-α-induced inhibition of osteoblast differentiation, and promote BMP-2-induced osteoblast differentiation. Whether GTX possesses NF-κB signal inhibiting activity was therefore investigated. Western blot analysis of NF-κB protein p65 was performed to detect whether GTX prevented p65 translocation to the nucleus. The results demonstrated that following treatment with 10 ng/ml TNF-α, p65 accumulated in the nucleus. However, no p65 accumulation was detected in the group that was also treated with 1 *μ*g/ml GTX (Fig. 5A). Subsequently, an immunofluorescent confocal microscopic assay for p65 localization detection was performed, and the results concurred that GTX prevented p65 accumulation in the nucleus (Fig. 5B). The luciferase assay method was also used to assess whether GTX attenuated TNF-α-induced NF-κB activity. The results suggested that 10 ng/ml TNF-α treatment induced a marked increase in NF-κB luciferase activity in C2C12 cells. The activation of luciferase activity was significantly reduced in the group that was treated with 0.5 *μ*g/ml GTX (P<0.05), suggesting that the activation of NF-κB was inhibited. This inhibition occurred in a GTX dose-dependent manner (Fig. 5C). These results indicated that GTX was able to markedly inhibit NF-κB signaling in C2C12 cells.

## Discussion

The inflammatory factor TNF-α has been demonstrated to have a critical role in the pathogenesis of bone-loss associated diseases. In the case of RA, TNF-α induces bone loss in the axial and appendicular skeleton (29). It was initially hypothesized that the promotion of osteoclast differentiation and bone resorption by TNF-α was significant in such cases of inflammatory bone loss; however, the inhibition of osteoblast differentiation by TNF-α was identified and required further investigation (7). Additionally, in estrogen deficiency-associated osteoporosis, TNF-α was found to be notably overproduced and may induce bone loss due to its biphasic effects in the promotion of bone resorption and reduction of bone formation (8,9).

NF-κB signaling is considered to have double effects in the development of osteoporosis, due to its bidirectional functions in bone resorption and formation (30). NF-κB responds to stimuli from receptor activator of nuclear factor κB ligand and directs osteoclast differentiation (31). Furthermore, it was identified to be an endogenous inhibitory factor for osteoblastic bone formation (13). Normal bone maintenance requires a balance between bone resorption and formation; therefore, the recovery of this balance is crucial for the effective treatment of osteoporosis. From this perspective, as biphasic factors, TNF-α and NF-κB may represent ideal therapeutic targets, and TNF-α and NF-κB inhibitors may represent potential therapeutic agents for the treatment of osteoporosis.

The fungi-derived secondary metabolite GTX has been demonstrated to be a potent NF-κB inhibitor, and is considered to be a potential agent for the treatment of immune glomerulo-nephritis (26). It was also demonstrated that GTX stimulated mature rat osteoclast apoptosis (32), and it was used as an experimental tool to inhibit NF-κB signaling in the study of osteoclastic gene regulations (33). However, whether GTX has a function in the modulation of osteoblast differentiation remained to be elucidated. In current study, the role of GTX in the regulation of osteoblast differentiation was evaluated by determining whether GTX was able to block the inhibition of BMP-2-induced differentiation by TNF-α and NF-κB. Based on the C2C12 osteoblast differentiation system, the results revealed that GTX protected ALP activity from TNF-α-induced impairment and rescued the promotion of *Col I*, *Ocn* and *Bsp* gene expression. Additional evidence from ALP staining and the expression of the three osteoblast-associated transcription factors, *Runx2*, *Osx* and *ATF4*, indicated that these osteoblastic characteristics were protected against the TNF-α-induced inhibition by GTX, which suggested that GTX rescued osteoblast differentiation by blocking the inhibitory effect exerted by TNF-α. The experiment also demonstrated that GTX promoted BMP-2-induced osteoblast differentiation, which indicated that GTX likely inhibited C2C12 endogenous NF-κB activity in order to facilitate osteoblast differentiation. Whether GTX was capable of blocking NF-κB activity in the C2C12 cell system was also evaluated. Western blot analyses and immunofluorescence confocal microscopic assays for the detection of p65 localization were performed and it was revealed that GTX prevented the accumulation of NF-κB protein p65 in the nucleus. Additionally, a luciferase activity assay was conducted in order to confirm that GTX inhibited the activation of NF-κB transcriptional activity, which suggested that GTX inhibited the activation of NF-κB signaling in C2C12 cells. In conclusion, GTX potentiates osteoblast differentiation by blocking the inhibition exerted by TNF-α and directly promotes BMP-2-induced osteoblast differentiation; these effects may be due to the inhibition of NF-κB signaling.

GTX was found to induce mature osteoclast apoptosis; therefore, it was hypothesized to have a potential anti-bone resorption function. The results of the present study suggested that GTX has protective and promotional effects on osteoblast differentiation and indicate that it may ultimately facilitate bone formation. Further studies are required to examine the potential role of GTX in regulating *in vivo* osteoblastic bone formation and treating bone loss diseases. Owing to the bidirectional function of GTX in the modulation of osteoclast and osteoblast effects, GTX may potentially be developed as a therapeutic agent to recover the bone resorption-formation balance and recover bone mass.
